# The putative Notch ligand HyJagged is a transmembrane protein present in all cell types of adult *Hydra *and upregulated at the boundary between bud and parent

**DOI:** 10.1186/1471-2121-12-38

**Published:** 2011-09-07

**Authors:** Andrea Prexl, Sandra Münder, Bernhard Loy, Elisabeth Kremmer, Susanne Tischer, Angelika Böttger

**Affiliations:** 1 Department of Biology 2, Ludwig-Maximilians-Universität München, Munich, Germany; 2 Institute for Neuroscience, Innsbruck Medical University, Innsbruck, Austria; 3 Institute for Molecular Immunology (IMI), Helmholtz Zentrum München, Munich, Germany

## Abstract

**Background:**

The Notch signalling pathway is conserved in pre-bilaterian animals. In the Cnidarian *Hydra *it is involved in interstitial stem cell differentiation and in boundary formation during budding. Experimental evidence suggests that in *Hydra *Notch is activated by presenilin through proteolytic cleavage at the S3 site as in all animals. However, the endogenous ligand for HvNotch has not been described yet.

**Results:**

We have cloned a cDNA from *Hydra*, which encodes a bona-fide Notch ligand with a conserved domain structure similar to that of Jagged-like Notch ligands from other animals. *Hyjagged *mRNA is undetectable in adult *Hydra *by *in situ *hybridisation but is strongly upregulated and easily visible at the border between bud and parent shortly before bud detachment. In contrast, HyJagged protein is found in all cell types of an adult hydra, where it localises to membranes and endosomes. Co-localisation experiments showed that it is present in the same cells as HvNotch, however not always in the same membrane structures.

**Conclusions:**

The putative Notch ligand HyJagged is conserved in Cnidarians. Together with HvNotch it may be involved in the formation of the parent-bud boundary in *Hydra*. Moreover, protein distribution of both, HvNotch receptor and HyJagged indicate a more widespread function for these two transmembrane proteins in the adult hydra, which may be regulated by additional factors, possibly involving endocytic pathways.

## Background

Early embryonic development is regulated in all animals by common signalling pathways such as the Wnt-, FGF-, Hedgehog-, TGFβ/BMP- and Notch pathways. They have been shown to be present in basal metazoans such as Cnidarians [1-5]. The fresh water polyp *Hydra *consists of a simple tube-like body made of two epithelial layers, the inner endoderm and the outer ectoderm separated by an acellular mesoglea. It further possesses a hypostome and a ring of tentacles at the apical end and a foot at its basal end. Interstitial cells are located in the spaces between the epithelial cells of both cell layers. They constitute a pluripotent stem cell lineage that differentiates nerve cells, gland cells, germ cells and nematocytes. The latter carry nematocyte capsules used for catching prey and are unique to Cnidarians [6-9].

The Notch signalling pathway has recently been shown to be involved in nematocyte and female germ cell differentiation, as well as in budding [5,10]. Whilst an inductive function of Notch for terminal differentiation of nematoblasts and oocytes has been suggested the precise mechanisms are completely unclear. During budding Notch signalling was shown to be important for the formation of a sharp boundary between the bud and the parent animal. This seemed to be the preposition for the bud to make a constriction at its base and form a foot. As has been shown at many other developmental boundaries Notch signalling here appears to have the potential to create patterns where two adjacent cells adopt different fates. These are signal receiving cells, which have the Notch receptor, and signal sending cells carrying a Notch ligand. Notch ligands possess a characteristic domain, the DSL (Delta, Serrate, Lag) domain, followed by several EGF repeats and a transmembrane domain. Serrate/Jagged-like ligands have an extra domain, the cysteine rich von Willebrand type C (VWC) domain, between the EGF repeats and the transmembrane domain. Interaction with Notch receptors takes place via the DSL domains of the ligand and the EGF repeats of the receptor [11].

We have now identified a potential Notch ligand in *Hydra*. Here we present its sequence and expression pattern. Comparison of its amino acid sequence with that of other Notch ligands of the DSL family shows similarity to Serrate/Jagged-like ligands. We thus named it HyJagged. Its mRNA expression levels in adult animals were not detectable by *in situ *hybridisation with the exception of a remarkable upregulation at final stages of budding in cells of the parent animal at the site where the bud detaches. However, immunofluorescence studies with anti-HyJagged antibody revealed the presence of HyJagged in all cells of adult animals where it is localised at membranes and in endosomes.

## Results

### Isolation and characterization of the *Hydra Jagged *gene

**Figure 1:**
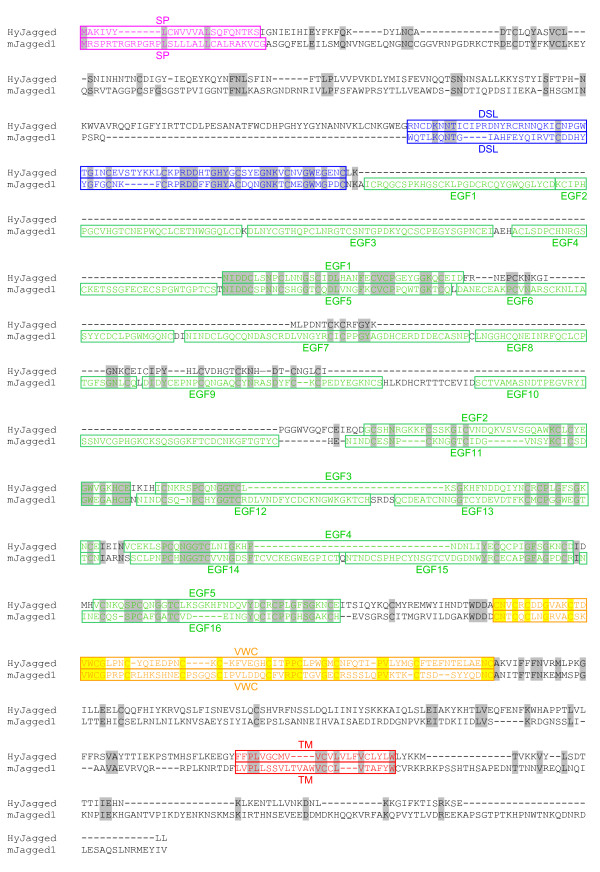
**Clustal X alignment of mJagged1 (mouse) and HyJagged (*Hydra*)**. Signal peptide (SP, pink), DSL domain (DSL, blue), EGF repeats (EGF, green), VWC domain (VWC, yellow), transmembrane domain (TM, red). Identical amino acids are highlighted in grey and yellow respectively.

**Figure 2:**
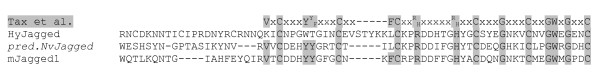
**Alignment of DSL domains from predicted NvJagged (*Nematostella*), mJagged1 (mouse) and HyJagged (*Hydra*) in comparison with the DSL domain consensus sequence suggested by Tax *et al*. **[13]. Consensus sequence of DSL domains according to [13]. Identical amino acids are marked in grey.

**Figure 3:**
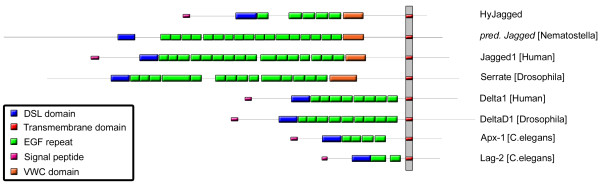
**Schematic domain structure of *Hydra *HyJagged in comparison with Delta and Serrate/Jagged-like proteins from *Nematostella*, *Caenorhabditis*, *Drosophila *and *Homo***. Signal peptide (pink), DSL domain (blue), EGF repeats (green), VWC domain (orange), transmembrane domain (red).

Database searches of the *Hydra *genome [12] led to a core sequence prediction for a DSL protein with similarity to Jagged/Serrate-like Notch ligands. After performing 5' and 3' RACE experiments the full-length *Hyjagged *sequence was amplified from *Hydra *cDNA [GenBank:JN036823]. The resulting HyJagged protein sequence is shown in Figure 1 in an alignment with mouse Jagged1. It contains a signal peptide (1-22aa), a DSL (Delta, Serrate, Lag) domain (181-252aa), which is characteristic for Notch ligands (Figure 2) [13], and five EGF repeats (255-540aa). A cysteine rich domain, the von Willebrand type C (VWC) domain (549-616aa), is located N-terminally of the transmembrane domain (763-785aa), followed by a short non-conserved intracellular region. Comparison with Notch ligands from worms, insects and vertebrates further shows that the modular structure is conserved in HyJagged (Figure 3). The presence of the von Willebrand type C domain, which is characteristic for Serrate/Jagged-like ligands, led us to name the *Hydra *protein HyJagged.

Interestingly, comparison of HyJagged with the predicted sequence of a Notch ligand from the sea anemone *Nematostella vectensis*, another Cnidarian, shows a generally similar domain structure between the two proteins but also an important difference (Figure 3). The *Nematostella *gene encodes a protein with 17 EGF repeats, whereas HyJagged has only six, which in both cases nearly matches the number of EGF repeats in their respective Notch receptors. The DSL domains between the Notch ligands from *Nematostella *and *Hydra *are also highly conserved (Figure 2).

**Figure 4:**
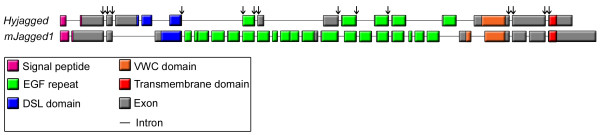
**Schematic representation of the exon-intron structure of *Hyjagged *(*Hydra*) in comparison to *mJagged1 *(mouse)**. Coding regions for the different protein domains are shown in colour: signal peptide (pink), DSL domain (blue), EGF repeats (green), VWC domain (orange), transmembrane domain (red), non-conserved exon regions (grey). Introns are not to scale (black lines). Conserved exon-intron-boundaries are marked with arrows.

HyJagged consists of 835 amino acids that are encoded by 16 exons on a stretch of 11.452 nucleotides. Comparison of the gene organisation of mouse *jagged1 *with *Hyjagged *is shown in Figure 4. All exon-intron boundaries are flanked by the conserved GT-AG sequences, 14 of these boundaries appear to be conserved between mouse and *Hydra *(Figure 4, indicated by arrows). These reflect the modular organisation of Jagged proteins in all animals. Most EGF repeats and the transmembrane domain are encoded by single exons. However the *Hydra *DSL domain and the mouse VWC domain, as well as the signal peptides of both proteins are encoded within two exons, respectively.

### mRNA expression of *Hyjagged*

To look for expression of the *Hyjagged *gene in adult hydra *in situ *hybridisation experiments were performed. These showed no signal, indicating that *Hyjagged *is expressed at very low levels in contrast to the expression of *HvNotch*, which shows very high levels of mRNA all over the animal [5]. This is in accordance with the fact that no ESTs encoding HyJagged could be identified in the libraries.

**Figure 5:**
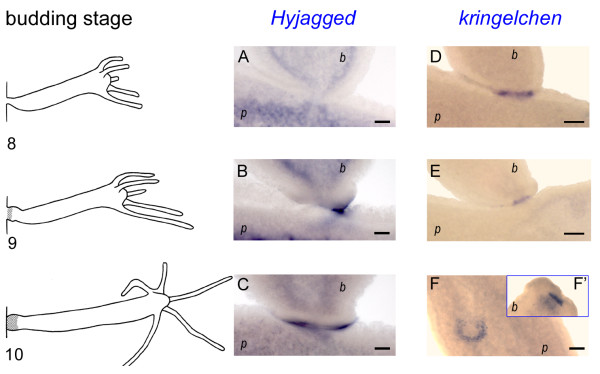
**Whole mount *in situ *hybridisation for *Hyjagged *and *kringelchen *during late budding stages**. Left hand panel: Budding stages 8, 9 and 10 according to [31]; (A-C) *in situ *hybridisation for *Hyjagged*, (D-F') *in situ *hybridisation for *kringelchen *from [10]; (F') foot of bud that fell off during staining procedure; (*p*) parent animal; (*b*) bud. NBT/BCIP (Roche) was used for staining reactions (blue signals). Scale bars: 20 μm.

However, when we looked for *Hyjagged *mRNA during budding we found a very strong expression of the gene during budding stage 9. This is when the bud begins to form foot cells. The signal was present in cells of the ectoderm of the parent animal and coincided with the expression zone of the *Hydra *FGF-receptor homolog *kringelchen *(Figure 5).

### Protein localisation of HyJagged

**Figure 6:**
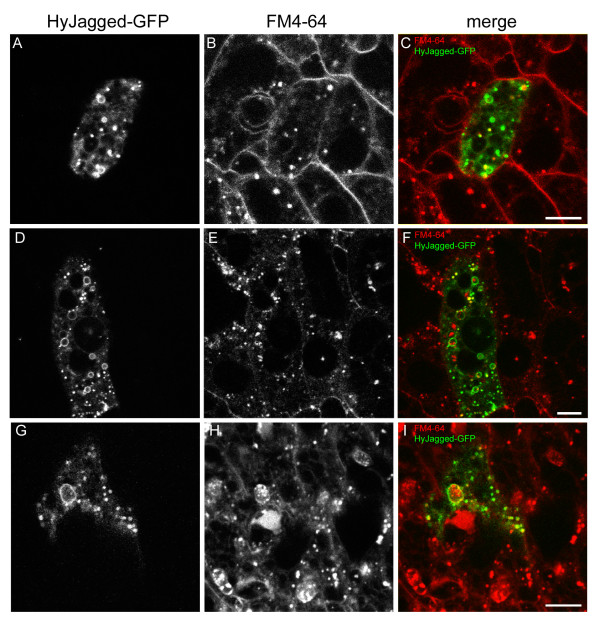
**Expression of HyJagged-GFP in *Hydra***. Animals with single HyJagged-GFP expressing cells after transfection with the particle gun were incubated with FM4-64 for 20 min (A-C), 1 h (D-F) and 20 h (G-I); (A, D, G) HyJagged-GFP in a single ectodermal epithelial cell; (B, E, H) FM4-64 in all cells of the animal; (C, F, I) merged images in false colours: HyJagged-GFP (green), FM4-64 (red); single confocal sections; scale bars: 10 μm.

**Figure 7:**
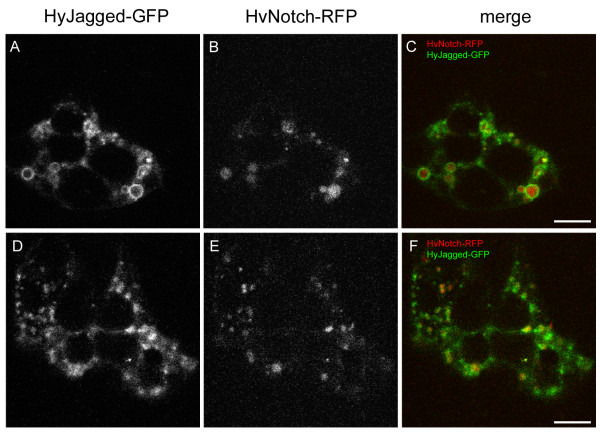
**Co-expression of HyJagged-GFP and HvNotch-RFP in *Hydra***. Two single ectodermal epithelial cells expressing both, HyJagged-GFP and HvNotch-RFP after transfection of animals with the particle gun (cell 1 A-C, cell 2 D-F). (A, D) HyJagged-GFP, (B, E) HvNotch-RFP, (C, F) merged images in false colours: HyJagged-GFP (green), HvNotch-RFP (red); single confocal sections; scale bars: 10 μm.

In order to study the subcellular distribution of HyJagged in hydra cells the protein was overexpressed as a GFP fusion protein in single cells of intact animals and observed *in vivo *(Figure 6). For detection of plasma membranes and endosomes the membrane-selective dye FM4-64 was injected into the gastric cavity and the animals were imaged at different time points afterwards. FM4-64 is usually detected at membranes first but is then increasingly enriched in endosomes, which become larger over time [14]. In Figure 6 we show the distribution of FM4-64 in cells of live imaged animals over a period of 20 hours. HyJagged-GFP hardly co-localised with FM4-64 at membranes at the time points indicated. However, it was seen in punctuate and ring-like structures in the cytoplasm. Some of these clearly corresponded to FM4-64 positive structures and thus most likely constituted early and late endosomes. We then compared this HyJagged-GFP distribution with the localisation of HvNotch-RFP in epithelial cells. Figure 7 shows that both proteins are localised in punctuate and ring-like structures, however they only partially co-localised.

In the absence of detectable mRNA for the putative Notch ligand *Hyjagged *we thought to look for the endogenous HyJagged protein. We therefore raised an antibody in rabbit against a synthetic peptide corresponding to 18 amino acids of the intracellular domain of HyJagged. The antibody recognised the intracellular domain of HyJagged when it was expressed in *E.coli *after SDS-PAGE and Western blotting (Additional file 1A). In addition, on a Western blot with a vesicle fraction from *Hydra *the antibody recognised a band at an apparent molecular weight of 130 kDa. This is larger than the HyJagged primary sequence suggests (96 kDa), most probably due to post-translational modification of mature HyJagged. Heavy glycosylation of EGF repeats is well documented for Notch ligands (recently reviewed in [15]).

**Figure 8:**
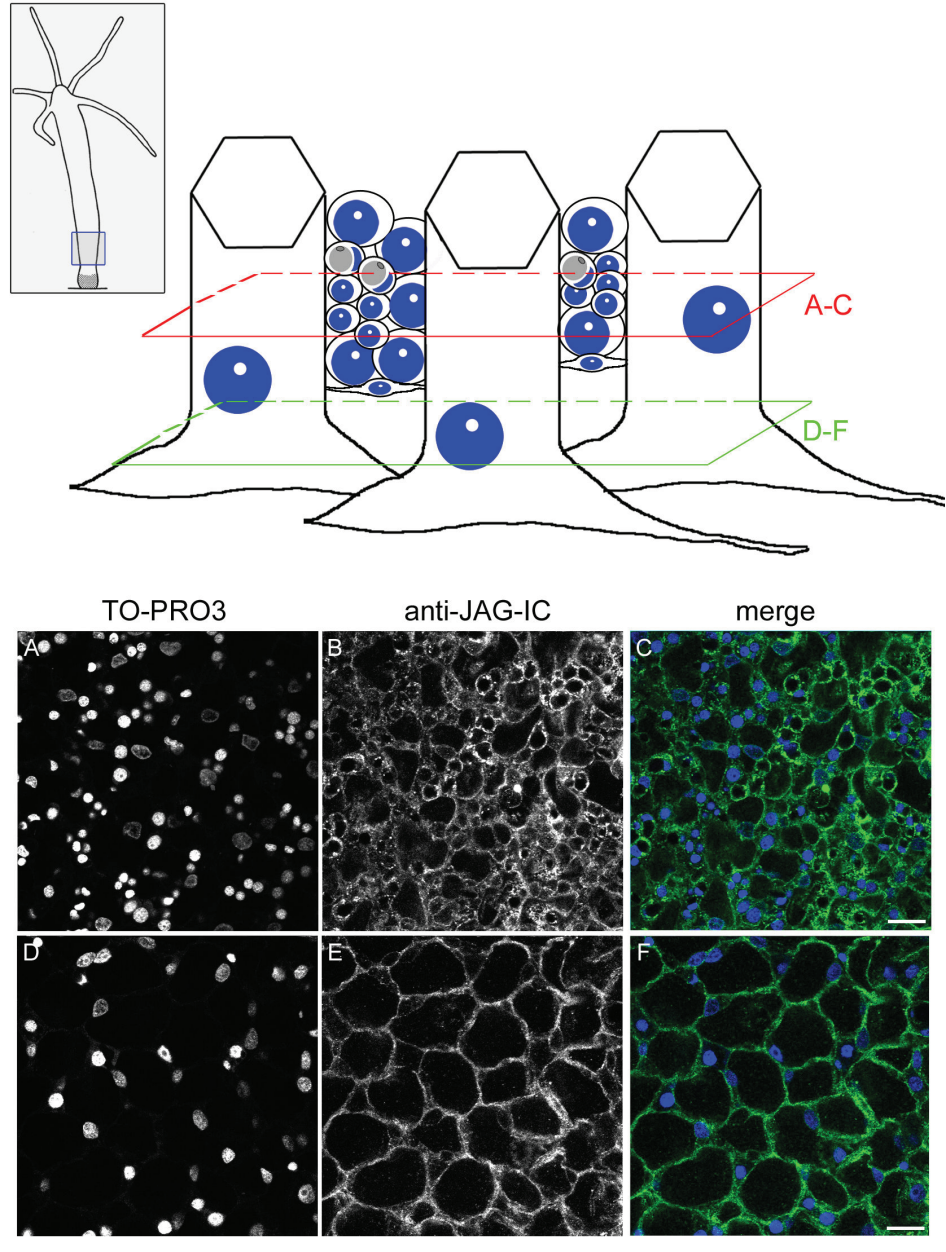
**Immunofluorescence of *Hydra *whole mounts with the anti-JAG-IC antibody**. Upper panel left: schematic representation of an adult hydra, the region imaged in lower panel is indicated, upper panel, right: schematic drawing of cells that were imaged including ectodermal epithelial cells, interstitial cells, nematoblasts, nematocytes and nerve cells; positions of confocal sections are boxed in red (A-C) and green (D-F). (A, D) DNA staining of single confocal sections with TO-PRO3, (B, E) anti-JAG-IC staining, (C, F) merged images in false colours: DNA (blue), anti-JAG-IC staining (green); scale bars: 20 μm.

**Figure 9:**
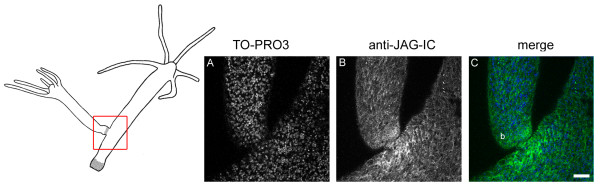
**Staining of HyJagged during budding**. Left hand panel: Schematic of an adult hydra with bud at stage 9, region imaged in A-C is indicated; (A) DNA staining of single confocal section with TO-PRO3, (B) anti-JAG-IC staining, (C) merged images of all channels in false colours: DNA (blue), anti-JAG-IC staining (green); scale bar: 50 μm.

The anti-JAG-IC antibody also detected HyJagged in immunofluorescence experiments, when we expressed it as GFP fusion protein in human HEK293T cells. This is shown in Additional file 1 D-G. In a transient transfection experiment in HEK293T cells HyJagged-GFP did not reach the membrane but was instead trapped in the ER as can be seen by strong staining of the nuclear envelope. This incorrect distribution of HyJagged is most probably due to incomplete post-translational processing of the hydra protein in human cells [16]. Nevertheless this experiment clearly shows that the antibody recognises HyJagged specifically in immunofluorescence. It did not react with any other human protein in HEK293T cells. We therefore used this antibody for immunofluorescence on *Hydra *whole mounts. Here it stained membranes and punctuate structures in the cytoplasm of epithelial cells all over the animal (Figure 8). A weak increase of the staining in comparison with the surrounding tissue was detected in budding animals at stage 9 at the base of the bud in the ectoderm of the parental animal (Figure 9). This is in accordance with the *in situ *hybridisation data, which indicated strong mRNA expression in this region.

**Figure 10:**
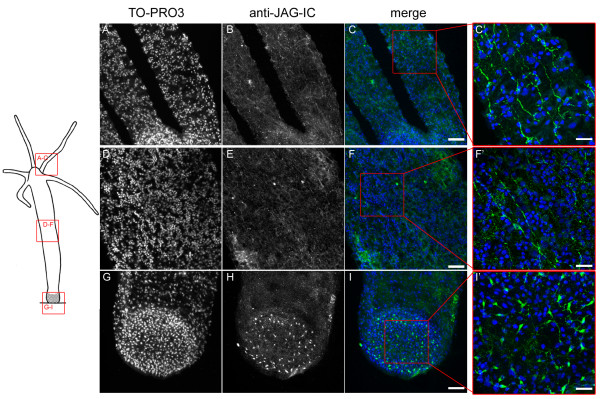
**Nerve cell staining of HyJagged**. Left hand panel: Schematic representation of an adult hydra; regions imaged in A-I are indicated; (A, D, G) DNA staining of stacks of confocal sections with TO-PRO3, (B, E, H) anti-JAG-IC staining, (C, F, I, C', F', I') merged images in false colours: DNA (blue), anti-JAG-IC staining (green); C'F' and I' are magnifications of C, F and I as indicated by red boxes; scale bars: (A-I) 50 μm, (C', F', I') 20 μm.

**Figure 11:**
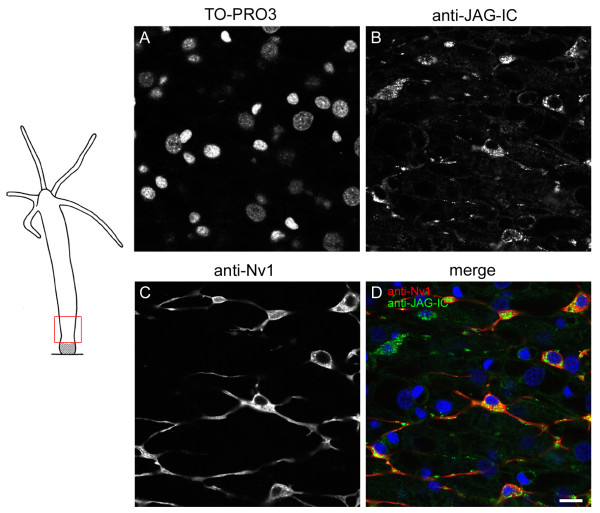
**Co-immunofluorescence staining with anti-JAG-IC- and anti-Nv1-antibodies**. Left hand panel: Schematic representation of an adult hydra with imaged region indicated; right hand panel: single confocal sections: (A) DNA staining with TO-PRO3, (B) anti-JAG-IC staining, (C) anti-Nv1 staining, (D) merged images of all channels in false colours: DNA (blue), anti-JAG-IC staining (green), anti-Nv1 staining (red); scale bar: 10 μm.

In addition to epithelial cells, cells of the interstitial cell lineage were also stained with the antibody. This was seen especially in nerve cells of the basal disc (Figure 10). Co-immunofluorescence experiments with the anti-JAG-IC- and anti-Nv1-antibodies were performed in order to relate HyJagged-positive cells to a specific cell type. The anti-Nv1-antibody is specific for sensory neurons in the tentacles and ganglion cells in the peduncle [17]. Co-staining with anti-JAG-IC showed that these ganglion cells also contained HyJagged, but in addition, HyJagged was also present in Nv-1 negative neurons (Figure 11).

**Figure 12:**
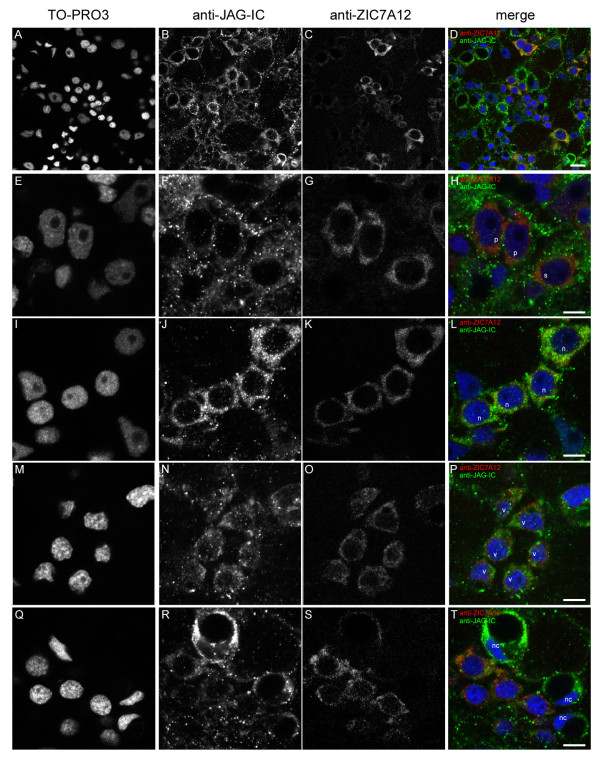
**Co-immunofluorescence staining with anti-JAG-IC- and anti-ZIC7A12-antibodies**. Single confocal sections of a hydra from a region of the body column: (A, E, I, M, Q) DNA staining with TO-PRO3, (B, F, J, N, R) anti-JAG-IC staining, (C, G, K, O, S) anti-ZIC7A12 staining, (D, H, L, P, T) merged images of all channels in false colours: DNA (blue), anti-JAG-IC staining (green), anti-ZIC7A12 staining (red); (A-D) overview of the staining pattern in the body column of an adult hydra, (E-H) localization of HyJagged in single (s) and a pair of interstitial cells (p), (I-L) staining of a nest of four nematoblasts (n), (M-P) nest of nematoblasts with vacuoles (v), (Q-T) staining of mature nematocytes (nc); scale bars: (A-D) 10 μm, (E-T) 5 μm.

In order to identify HyJagged proteins in cells of the nematocyte line we used a rat monoclonal antibody against HyZic protein since *Hyzic *mRNA is known to be expressed in proliferating nematocyte precursors [18]. Rats were immunized with HyZic protein that we produced in *E.coli*. The resulting monoclonal antibody anti-ZIC7A12 detected the recombinant protein on Western blots. Moreoever, it stained a band of 46 kDa, which corresponds to the predicted size of HyZic in Western blots from hydra lysates (Additional file 2). In addition, in immunofluorescence experiments it recognised a HyZic-GFP fusion protein when expressed in hydra cells (see Additional file 2). On *Hydra *whole mounts the anti-ZIC7A12 antibody strongly stained single and double interstitial cells and nests of nematoblasts before their exit from mitosis. Weaker staining was observed in post-mitotic nematoblasts, which already had formed a small vacuole. In further developed nematocytes HyZic was not detected anymore. This distribution of HyZic protein in *Hydra *is in good agreement with published data about the localisation of its mRNA in proliferating nematoblasts [18]. Figure 12 shows that single and pairs of interstitial cells highlighted with the anti-ZIC7A12 antibody also contained HyJagged in a punctuate pattern. This was also the case in proliferating nematoblasts. However, in finally differentiated nematocytes negative for HyZic very strong HyJagged staining was observed (Figure 12 Q-T, nc). In conclusion, while all cells of the nematocyte line seem to have HyJagged protein, its abundance increases in terminally differentiated cells.

**Figure 13:**
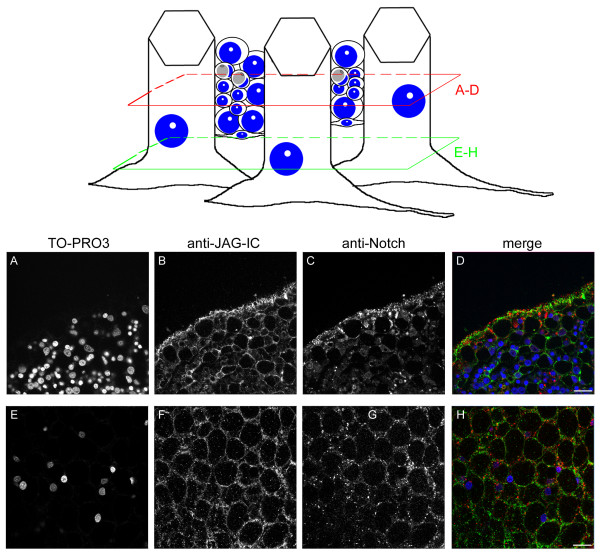
**Co-immunofluorescence staining of *Hydra *whole mount with anti-JAG-IC- and anti-Notch-antibodies**. Upper panel left: schematic representation of an adult hydra, the region imaged in lower panel is indicated; upper panel, right: schematic drawing of cells that were imaged including ectodermal epithelial cells, interstitial cells, nematoblasts, nematocytes and nerve cells; positions of confocal sections are boxed in red (A-D) and green (E-H). (A, E) DNA staining with TO-PRO3, (B, F) anti-JAG-IC staining, (C, G) anti-Notch staining, (D, H) merged images in false colours: DNA (blue), anti-JAG-IC staining (green), anti-Notch staining (red); single confocal sections; scale bars: 20 μm.

Finally, we wanted to compare the cellular localisation of HyJagged with that of HvNotch. To this end we raised an antibody in chicken against HvN^ΔE ^(the *Hydra *Notch receptor HvNotch without its extracellular domain) after its expression in *E.coli*. It detected HvN^ΔE ^after expression in *E.coli *by SDS-PAGE and Western blotting down to a concentration of 8, 9 ng (see Additional file 3A). Furthermore it recognised overexpressed HvNotch-GFP fusion protein in human HEK293T cells in immunofluorescence experiments (see Additional file 3). We thus used it for co-immunofluorescence staining on *Hydra *whole mounts together with the anti-JAG-IC antibody. Both antibodies stained membranes of epithelial cells and cells in the interstitial spaces, however, the patterns did not completely overlap. Moreover, both antibodies stained punctuate structures in the cytoplasm. From co-localisation experiments of live images where HvNotch and HyJagged clearly co-localised with the membrane selective dye FM4-64 we concluded that these punctae most likely correspond to endosomes. However, HyJagged- and HvNotch antibody signals in endosomes do not generally co-localise. Moreover, HyJagged signals appear stronger localised on the membranes in comparison with Notch, this is especially obvious in interstitial cells (Figure 13).

## Discussion

In this work we have identified a potential ligand for HvNotch in *Hydra*, HyJagged. It has the same modular structure as Notch ligands from higher animals and the closely related Cnidarian *Nematostella*, which is also reflected in the conserved exon-intron structure of its gene between *Hydra *and mouse. The protein includes a DSL domain, which is responsible for the interaction with the extracellular EGF repeats of the Notch receptors in flies and mammals [11]. Moreover, HyJagged possesses a relatively small number of EGF repeats and a transmembrane domain. Comparison of the abundance of EGF repeats in Notch ligands and receptors shows some interesting similarities: Species with large extracellular domains in their Notch receptors (e.g. *Nematostella*, *Drosophila *and mouse) also have a high number of EGF repeats in their Serrate/Jagged-like proteins (however not in Delta) and vice versa (Figure 3 and [5]).

Thus, although we have not provided experimental evidence, we suggest that HyJagged constitutes the only canonical Notch ligand in *Hydra*. A second gene encoding a DSL domain is also present in *Hydra*. It has been identified previously and was shown to be expressed in gland cells [19]. The resulting protein is 332 amino acids long, has a signal peptide and a DSL domain and thus shows some structural homology with DSL2, a soluble Notch ligand from *C.elegans*. It would be interesting to see whether this protein is involved in Notch signalling in *Hydra*.

*In situ *hybridisation showed that *Hyjagged *was not visibly expressed on the mRNA level in intact *Hydra *with the exception of a very strong upregulation at budding stage 9. We have previously described that Notch signalling is important for late budding stages in *Hydra*. Whilst as early as at budding stage 5-6 HvWnt8 and the *Hydra *FGFR-homolog *kringelchen *demarcate the boundary between the parent animal and the bud [20,21] budding cannot be completed without Notch signalling. The gene expression zone of *kringelchen *remains diffuse and a crucial metalloprotease, MMP-A3 is not induced. Thus the bud cannot constrict and does not form a foot. Two stripes of gene expression are required for correct patterning of the bud, one leading to constriction involving MMP-A3 and the other leading to differentiation of the buds foot cells involving expression of foot specific genes such as *ppod-1*. For these to form we proposed a model including mutual inhibition and lateral activation on the basis of Notch signalling at this boundary [10].

Our model required that cells which express *kringelchen *at the boundary express a Notch ligand. The data shown here are partially in accordance with this assumption because *Hyjagged *is expressed in cells that also express *kringelchen *at the boundary between parent and bud. However, the timing of this upregulation is unexpected. While constriction begins in budding stage 8, *Hyjagged *mRNA could only be detected at the beginning of budding stage 9. *Hyjagged *expression during earlier budding stages probably occurs in too low amounts to be detectable by *in situ *hybridisation experiments. Antibody staining clearly shows the presence of HyJagged at the basis of buds, however, only a weak increase in its level at this position in comparison with the remaining tissue is detectable (Figure 9). At this point we clearly have not used techniques suited to uncover precise quantitative differences in the amounts of HyJagged mRNA and protein at the cellular level during the final stages of budding. Therefore we cannot exclude the possibility that the observed requirement for Notch signalling at the boundary between parent and bud involves a mechanism that does not include a direct HyJagged-HvNotch interaction.

In contrast to the generally undetectable levels of mRNA expression outside the boundary between parent and bud, HyJagged protein is clearly present in all cells of the animals. The same is true for HvNotch. Although this makes it difficult to conclude whether there is active Notch signalling going on, the presence of the proteins is not unexpected. Notch signalling is highly regulated by factors involved in post-translational modification of the receptors (e.g. Fringe) [22], by the activity of metalloproteases like ADAM [23] and of presenilin as catalytic subunit of the γ-secretase complex and also by endocytosis of both, the Notch receptors and the ligands [24,25]. Endocytosis is part of a mechanism that leads to mutual inhibition of the signal sending and signal receiving cells. This means that signalling cells have higher levels of ligand but inhibit the propagation of Notch signals from adjacent cells and vice versa [26]. That way signalling can be induced by small differences in the concentration of receptors and ligands in this system, which might be hard to detect in immunofluorescence experiments [27]. We have shown that GFP/RFP-tagged HyJagged and HvNotch are indeed localised in endosomes (this work and [5]). However, the fluorescently tagged protein did not completely co-localise with each other within these structures. Differences in the localisation of the endogeneous proteins were also seen in immunofluorescence experiments. HyJagged appeared stronger localised at membranes whereas HvNotch showed a slightly stronger staining of punctae in the vicinity of the membranes. In interstitial cells membrane staining was completely lacking with the HvNotch antibody.

In *Hydra*, Notch signalling might also be regulated through post-translational modification of receptor and ligand as genes encoding metalloproteases of the ADAM family, the glycosyltransferase *Fringe *and the ubiquitin ligase *Mindbomb*, which regulates endocytosis of Notch ligands, are present in the genome [12]. Their expression patterns might be conclusive in the future in order to understand the precise molecular mechanism by which Notch signalling exerts its profound impact on pattern formation and stem cell differentiation in *Hydra*.

## Conclusions

We report here the identification of the *Hyjagged *gene, which encodes a protein with great similarity to canonical Notch ligands from worms, insects and vertebrates. Its mRNA is especially strongly expressed at the constriction site of budding animals, confirming the conserved role for Notch ligands during boundary formation. Furthermore, both HvNotch and HyJagged proteins are found in all cells of adult animals indicating a general role for Notch signalling in regulating cellular communication in *Hydra*. The differential distribution of both proteins in endosomes indicates that endosomal pathways may play an important role in modulating Notch signalling.

## Methods

### Hydra culture

*Hydra vulgaris *(Basel) was grown at a temperature of 18°C in hydra medium (0.4 mM CaCl_2_, 0.6 mM MgSO_4_, 0.5 mM NaHCO_3_, 0.08 mM K_2_CO_3_). The animals were fed regularly with freshly hatched *Artemia nauplii*.

### PCR amplification of *Hyjagged *sequences

*Hydra *cDNA for RACE experiments was obtained with the GeneRacer Kit with Superscript III (Invitrogen). 5' and 3'RACE experiments were performed using the gene specific primers 5'-CCCATTTGTT GTGAGGCGTA AAGCTGATGT AAGTGC-3' and 5'-GGAGTTGCAA AATGTACAGA TGCATGGTGT GG-3'. Full-length *Hyjagged *was then amplified from *Hydra *cDNA by PCR.

### Whole mount *in situ *hybridisation experiments

Single whole mount *in situ *hybridisation experiments were carried out using Digoxigenin-labeled RNA probes as described before [17,28].

### Construction of GFP and RFP fusion proteins

Full-length *Hvnotch *was cloned into the NheI and SmaI sites of the HoTRed expression vector. Full-length *Hyjagged *as well as *Hyzic *were cloned into the modified EGFP expression vector HoTG(reen) [29] into the NheI and SmaI sites for overexpression from the *Hydra *actin promoter in hydra cells.

For expression in mammalian cells *HvNotch *and *Hyjagged *genes were cloned into the EcoRI and SmaI sites of the expression vector pEGFP (Clontech), leading to expression of C-terminally GFP-tagged proteins.

### Transfection of Hydra cells

GFP- and RFP-constructs were introduced into hydra cells using a particle gun (Biorad) as described previously [29]. After 1-2 days cells expressing the GFP and RFP fusion proteins were clearly visible.

### Production of recombinant proteins

To allow expression in *E.coli *the coding sequences for *HvN^ΔE ^*(HvNotch without its extracellular domain), *HvNICD *, *Hyjagged-ICD*, *Hyzic, HyHes and Cnash *were cloned into the vector pRSET (Invitrogen) using the BamHI and EcoRI sites for *HvN^ΔE^, HvNICD, Hyjagged-IC and Hyzic*, and the EcoRI and XhoI sites for *Cnash and HyHes*.

Denatured His_6_-tagged-HyJagged-ICD, -HvN^ΔE ^, -HvNICD, -HyZic, -HyHES and -CnASH were produced in *E.coli *and affinity-purified using nickel sepharose (Amersham Biosciences). Recombinant HvN^ΔE ^and HyZic were used for antibody production, recombinant HyJagged-ICD, HvNICD, HyZic, HyHES and CnASH were analysed by SDS-PAGE and Western Blot using anti-Notch (dilution 1:100), anti-JAG-IC (dilution 1:500), anti-ZIC7A12 (dilution 1:1), anti-HES (dilution 1:500) and anti-CNA7B10 (dilution 1:1) antibodies.

### Antibody production

Anti-Notch antibody was produced in chicken by Davids Biotechnologie GmbH by immunisation with recombinant HvN^ΔE^. A portion of the resulting polyclonal antibody was then affinity purified.

For the anti-JAG-IC antibody a peptide corresponding to a stretch of the intracellular domain of HyJagged (VNKDNLKKGIFKTISRKS) was produced by Davids Biotechnologie GmbH and used for immunisation of rabbit. The resulting antibody was then affinity purified.

For the anti-HES antibody a peptide corresponding to a stretch of the region shortly before and the begin of the basic domain of HyHES (RHPMKEKRRANKPLLER) was produced by Davids Biotechnologie GmbH and used for immunisation of rabbit. The resulting antibody was then affinity purified.

Monoclonal anti-ZIC7A12- and anti-CNA7B10-antibodies were produced in rat as described in [30]. Recombinant HyZic and CnASH were used for immunisation.

### Cultivation and transfection of HEK293T

Human embryonic kidney cells (293T) cells were cultured in DMEM (Sigma) supplemented with 10% FCS and 5% Pen/Strep at 37°C and 5% CO_2_. 293T cells were transfected using PEI pH 7,0.

### Antibody staining of HEK293T

HEK293T cells at 70-80% confluence were seeded on glass coverslips in 6-well culture plates and incubated for 24 h at 37°C. After that the cells were transfected with the specific DNA. After 24 h the coverslips were washed twice with PBS, and the cells were fixed in 4% PFA in PBS for 15 minutes at room temperature. After washing the cells again, they were permeabilized for 15 minutes with 1% Triton X-100 in PBS, washed twice with PBS and incubated in blocking solution (10% FCS, 0,2% Tween 20, PBS) for one hour at room temperature. Cells were then incubated with primary antibody (anti-Notch antibody (dilution 1:100) or anti-JAG-IC antibody (dilution 1:50)) for further 60 minutes at room temperature. After that, they were washed three times for 10 minutes in washing solution (1% BSA, 0,2% Tween 20, PBS) before they were incubated in secondary antibody for one hour at room temperature. Finally, cells were washed three times for 10 minutes with washing solution, incubated with TO-PRO 3 (Molecular probes, 1 μg/ml in PBS) for 5 minutes and mounted on slides with Vectashield (Alexis Biochemicals).

### Subcellular fractionation

500 hydra were dissociated into single cells in dissociation medium (3.6 mM KCl; 6 mM CaCl_2_; 1.2 mM MgSO_4_; 6 mM sodium citrate; 6 mM sodium pyruvate; 6 mM glucose; 12.5 mM TES; and 50 mg/ml rifampicin, pH6.9) by pipetting. After centrifugation the resulting cell pellet was resuspended and incubated for 4 hours at 18°C on a rotator in 20 μM of the proteasome inhibitor MG-132 (Sigma). The cells were then incubated on ice in 500 mM Saccharose, 10 mM Tris, 2 mM EGTA pH7,4, 10 ng/ml Pepstatin A, 10 ng/ml Aprotinin, 10 ng/ml Leupeptin, 0,5 mg/ml Pefablock for 20 min before homogenization with a Potter-Dounce homogenizer. The homogenate was differentially centrifuged. The 1000 g and 100.000 g pellets were separated by SDS-PAGE and Western Blots were stained with the anti-JAG-IC (dilution 1:500), rabbit preimmune serum (1:1000), anti-HES (dilution 1:500), anti-ZIC7A12 (dilution1:1) or anti-CNA7B10 (dilution1:1) respectively.

### Antibody staining of whole *Hydra*

For membrane staining of endogenous HvNotch and endogenous HyJagged whole animals were relaxed with 2% urethane and fixed with PFA/EtOH (2% PFA, 70% EtOH, PBS) for 30 minutes. They were then washed twice for 5 minutes with PBS followed by an ethanol treatment. Therefore hydra were treated with 20%, 30%, 40%, 50%, 75% EtOH in PBS and 100% EtOH, followed by 75%, 50%, 40%, 30% and 20% EtOH in PBS, each for 5 minutes. Further staining was performed as below, starting with permeabilization.

Other immunofluorescence staining experiments were performed by relaxing animals with 2% urethane and fixation with 4% PFA in hydra medium for 1 hour. Hydra were then washed three times for 10 minutes with PBS before they were permeabilized for 15 minutes with 0,5% Triton X-100 in PBS. Animals were blocked for 15 minutes with 0,1% Triton X-100, 1% BSA in PBS and incubated in anti-Notch antibody (dilution 1:100), anti-JAG-IC antibody (dilution 1:50), anti-Nv1 (dilution 1:2) or anti-ZIC7A12 (dilution 1:1) for 1 h at room temperature. Hydra were washed three times 10 minutes in PBS followed by incubation in secondary antibody for 2 h at room temperature. Before the animals were stained with TO-PRO3 (Molecular probes, 1 μg/ml in PBS) they were washed in PBS three times 10 minutes. Finally they were mounted on slides with Vectashield (Alexis Biochemicals).

### Confocal imaging

Living animals with GFP-expressing cells were stained with FM4-64 (Molecular Probes) for detection of plasma membranes and endosomes. 1 μl of 500 μM FM4-64 in hydra medium was injected into the gastric cavity of *Hydra*. The animals were then incubated in 50 μM FM4-64 in hydra medium for 20 minutes to 20 hours.

Living animals with GFP- and RFP-expressing cells were relaxed in 2% urethane and scanned immediately.

Light optical serial sections were acquired with a Leica (Leica Microsystems, Wetzlar, Germany) TCS SP confocal laser-scanning microscope equipped with an oil immersion Plan-Apochromat 100/1.4 NA objective lens. Fluorochromes were visualized with an argon laser with an excitation wavelength of 488 nm and emission filters of 520-540 nm for EGFP and Alexa488 and 640-760 nm for FM4-64, and with a helium-neon laser with an excitation wavelength of 633 nm and emission filter of 660-760 nm for TO-PRO3. For Cy3 and RFP the excitation wavelength was 561 nm and an emission filter of 570-580 nm was used. Image resolution was 512 × 512 pixel. The 8-bit grey scale single-channel images were overlaid to an RGB image assigning false color to each channel, and then assembled into tables using Adobe Photoshop 10.0 and ImageJ 1.37 k software.

### Accession numbers

GenBank accession numbers for genes and proteins used in alignments and domain structures are as follows:

HyJagged (JN036823); Mouse Jagged1 (NP_038850); Human Jagged1 (AAC51731); Human Delta-like1 (NP_005609); *Drosophila *Serrate (CAA40148); *Drosophila *DeltaD1 (AAR21456); *C.elegans *Apx-1 (NP_503882); *C.elegans *Lag-2 (NP_503877); *Hydra *HvNotch (ABV68547).

## Authors' contributions

AP isolated and characterised the *Hydra *Jagged homolog and carried out phylogenetic analysis, *in situ *hybridisations and protein localization studies. SM participated in *in situ *hybridisation, immunofluorescence experiments and Western blotting of subcellular fractions. EK produced the anti-ZIC7A12 monoclonal antibody. BL produced the protein for immunization of chicken and tested the anti-Notch antibody. ST participated in subcellular fractionation and *in situ *hybridisation. AB conceived and coordinated the study. AP and AB drafted the manuscript. All authors read and approved it.

## Supplementary Material

Additional file 1 **Testing of rabbit anti-JAG-IC peptide antibody**. (A-C) Western Blot after SDS-PAGE with bacterial lysates from *E.coli *expressing HyJagged-ICD (10 kDa) from pRSET (lanes labelled R JAG-IC 250 ng) and with vesicle fraction from hydra homogenates of 125 animals (lanes labelled V 125 a) probed with anti-JAG-IC antibody (A); Control with rabbit pre-immune serum (B); (C) Control Western blot probed with an unrelated rabbit antibody. This antibody had been raised against a peptide derived from Hydra HES and recognises HyHES (25 kDa) in bacterial lysates from *E.coli *expressing HyHES from pREST (lane RHyHES 50 ng). (D-G) HEK293T cells expressing HyJagged-GFP from pcDNA3; (D) DNA staining with TO-PRO3, (E) HyJagged-GFP, (F) anti-JAG-IC staining, (G) merged images in false colours: DNA (blue), HyJagged-GFP (green), anti-JAG-IC staining (red); Confocal sections; scale bar: 5 μm. Click here for file

Additional file 2 **Characterization of anti-ZIC7A12 antibody**. (A, B) Western blot after SDS-PAGE of bacterial lysates expressing HyZic from pRSET (lanes recomb. HyZic 50 ng and 10 ng) and lysates from hydra cells (lanes Hydra Ly) probed with monoclonal rat antibody anti-ZIC7A12 (A). Control with a rat IgG that had been raised against CnASH (B). This antibody recognises a band below 43 kDa in hydra lysates. The anti-ZIC7A12 recognises a band at 46 kDa, which corresponds to the predicted size for HyZic. (C-F) Single hydra epithelial cell ectopically expressing HyZic-GFP fusion protein from HotG after transfection of the animals with particle gun. (C) DNA staining with TO-PRO3, (D) HyZic-GFP, (E) anti-ZIC7A12 staining, (F) merged images in false colours: DNA (blue), HyZic-GFP (green), anti-ZIC7A12 staining (red); Confocal sections; scale bar: 2 μm. Click here for file

Additional file 3 **Characterisation of anti-Notch antibody**. (A-C) Western Blot after SDS-PAGE of lysates from *E.coli *expressing myc tagged HvN^ΔE ^(49 kDa) from pRSET probed with anti-Notch antibody (A), probed with chicken preimmune serum (B), probed with anti-myc antibody (C); arrows indicate HvN^ΔE ^and two apparent degradation products; (D-G) HEK293T cells expressing HvNotch-GFP from pcDNA3. (D) DNA staining with TO-PRO3, (E) HvNotch-GFP, (F) anti-Notch antibody staining (G) merged images in false colours: DNA (blue), HvNotch-GFP (green), anti-Notch staining (red); Confocal sections; scale bar: 5 μm. Click here for file
