# Supplementary figures and images for: The putative Notch ligand HyJagged is a transmembrane protein present in all cell types of adult Hydra and upregulated at the boundary between bud and parent
Source: BMC Cell Biol. 2011 Sep 7;12:38. doi: 10.1186/1471-2121-12-38 (PMC3180645; doi:10.1186/1471-2121-12-38)

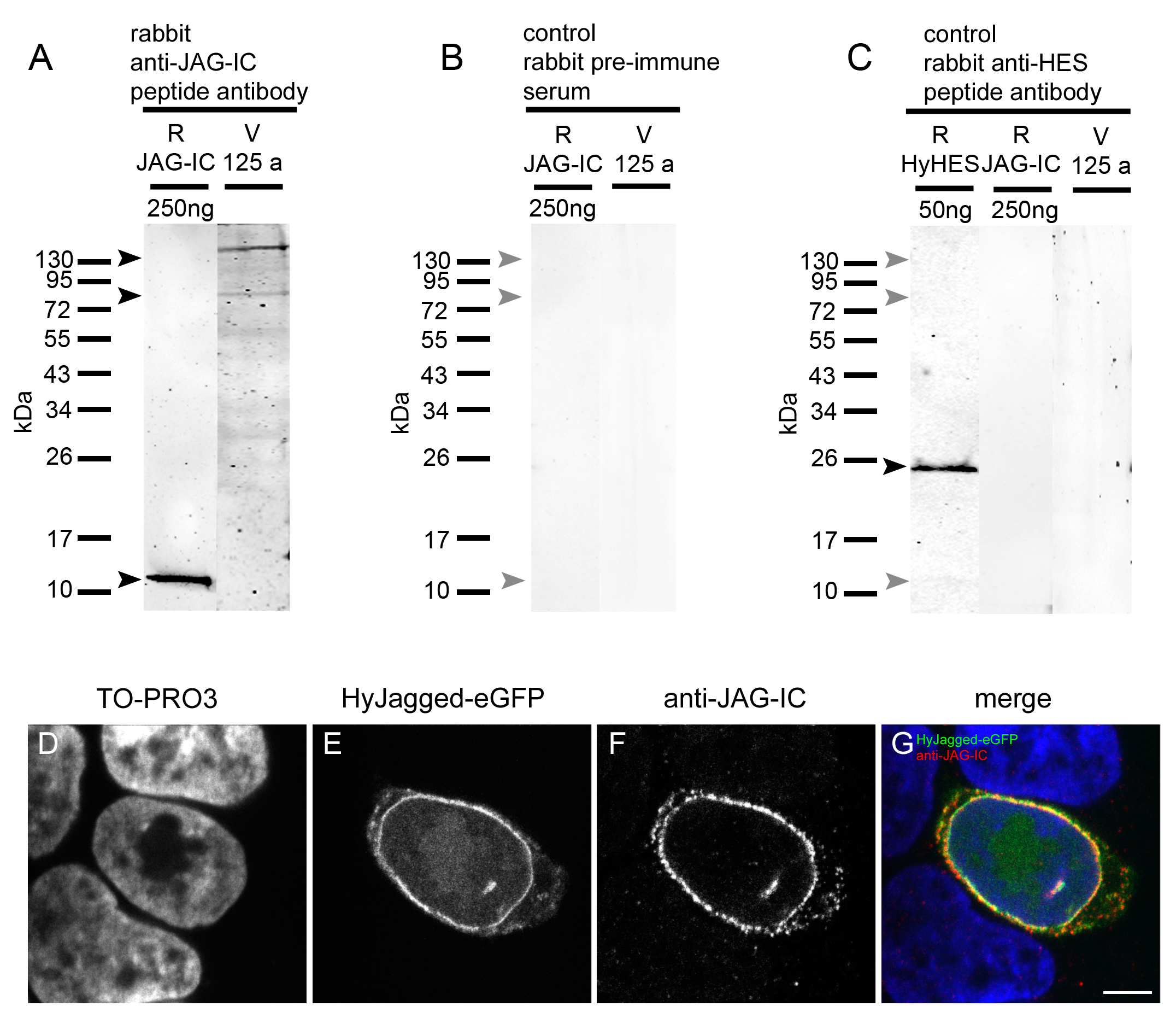

Supplement: Additional file 1 — Testing of rabbit anti-JAG-IC peptide antibody. (A-C) Western Blot after SDS-PAGE with bacterial lysates from E.coli expressing HyJagged-ICD (10 kDa) from pRSET (lanes labelled R JAG-IC 250 ng) and with vesicle fraction from hydra homogenates of 125 animals (lanes labelled V 125 a) probed with anti-JAG-IC antibody (A); Control with rabbit pre-immune serum (B); (C) Control Western blot probed with an unrelated rabbit antibody. This antibody had been raised against a peptide derived from Hydra HES and recognises HyHES (25 kDa) in bacterial lysates from E.coli expressing HyHES from pREST (lane RHyHES 50 ng). (D-G) HEK293T cells expressing HyJagged-GFP from pcDNA3; (D) DNA staining with TO-PRO3, (E) HyJagged-GFP, (F) anti-JAG-IC staining, (G) merged images in false colours: DNA (blue), HyJagged-GFP (green), anti-JAG-IC staining (red); Confocal sections; scale bar: 5 μm. [file 1471-2121-12-38-S1.TIFF]

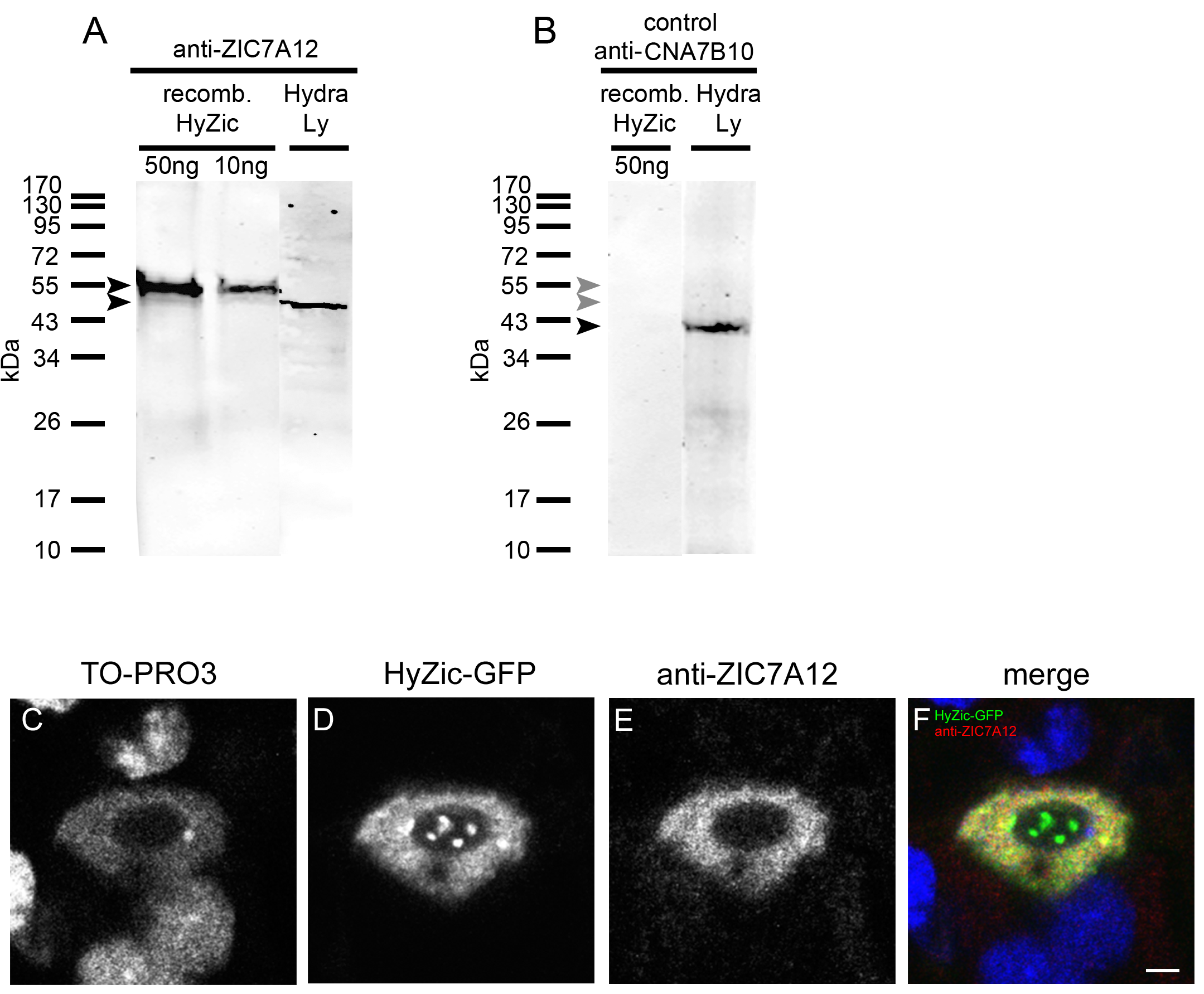

Supplement: Additional file 2 — Characterization of anti-ZIC7A12 antibody. (A, B) Western blot after SDS-PAGE of bacterial lysates expressing HyZic from pRSET (lanes recomb. HyZic 50 ng and 10 ng) and lysates from hydra cells (lanes Hydra Ly) probed with monoclonal rat antibody anti-ZIC7A12 (A). Control with a rat IgG that had been raised against CnASH (B). This antibody recognises a band below 43 kDa in hydra lysates. The anti-ZIC7A12 recognises a band at 46 kDa, which corresponds to the predicted size for HyZic. (C-F) Single hydra epithelial cell ectopically expressing HyZic-GFP fusion protein from HotG after transfection of the animals with particle gun. (C) DNA staining with TO-PRO3, (D) HyZic-GFP, (E) anti-ZIC7A12 staining, (F) merged images in false colours: DNA (blue), HyZic-GFP (green), anti-ZIC7A12 staining (red); Confocal sections; scale bar: 2 μm. [file 1471-2121-12-38-S2.TIFF]

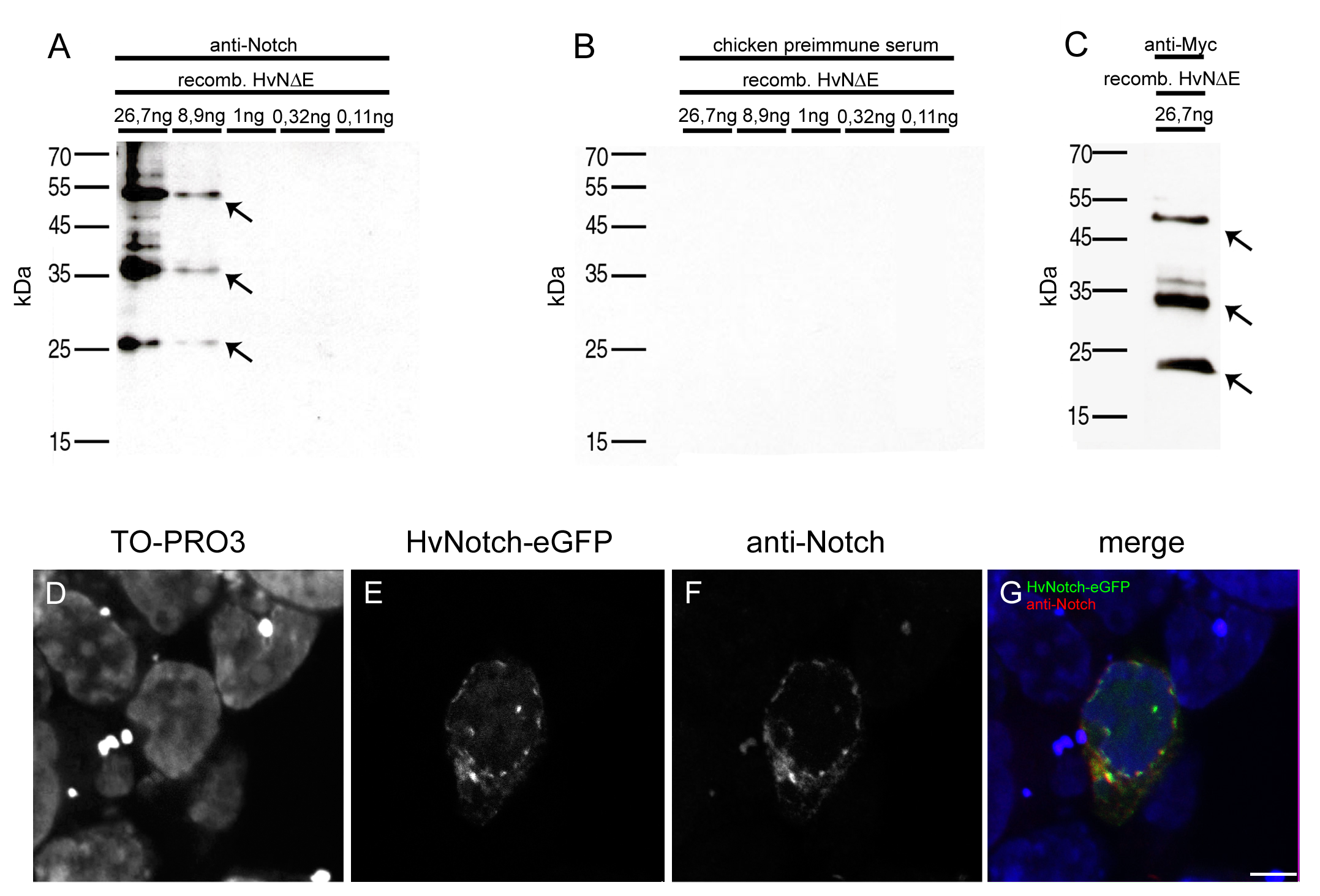

Supplement: Additional file 3 — Characterisation of anti-Notch antibody. (A-C) Western Blot after SDS-PAGE of lysates from E.coli expressing myc tagged HvNΔE (49 kDa) from pRSET probed with anti-Notch antibody (A), probed with chicken preimmune serum (B), probed with anti-myc antibody (C); arrows indicate HvNΔE and two apparent degradation products; (D-G) HEK293T cells expressing HvNotch-GFP from pcDNA3. (D) DNA staining with TO-PRO3, (E) HvNotch-GFP, (F) anti-Notch antibody staining (G) merged images in false colours: DNA (blue), HvNotch-GFP (green), anti-Notch staining (red); Confocal sections; scale bar: 5 μm. [file 1471-2121-12-38-S3.TIFF]
